# Is Artificial Intelligence Ready for Emergency Department Triage? A Retrospective Evaluation of Multiple Large Language Models in 39,375 Patients at a University Emergency Department

**DOI:** 10.3390/jcm15041512

**Published:** 2026-02-14

**Authors:** Ioannis Nedos, Sofia-Chrysovalantou Zagalioti, Christos Kofos, Theoni Katsikidou, Dimitra Vellidou, Konstantinos Astrinakis, Ioannis Karagiannis, Panagiotis Giannakopoulos, Styliani Michaloudi, Aikaterini Apostolopoulou, Efstratios Karagiannidis, Barbara Fyntanidou

**Affiliations:** Department of Emergency Medicine, AHEPA University General Hospital, Aristotle University of Thessaloniki, 541 24 Thessaloniki, Greece; nedos.ioannis@gmail.com (I.N.); sofia_zag@yahoo.com (S.-C.Z.); chriskofos21@gmail.com (C.K.); theonikatsikidou@gmail.com (T.K.); vellidoyd@gmail.com (D.V.); kostas.astrinakis7@gmail.com (K.A.); johnkaragiannis2002@gmail.com (I.K.); panagiot.giann@gmail.com (P.G.); stellamichaloudi@gmail.com (S.M.); katapost@yahoo.gr (A.A.); bfyntan@yahoo.com (B.F.)

**Keywords:** large language models, emergency triage, clinical decision support, artificial intelligence, emergency medicine

## Abstract

**Background:** Large language models (LLMs) are increasingly proposed as clinical decision support tools. However, their reliability in the emergency department (ED) triage remains insufficiently validated. This study aimed to evaluate the performance and limitations of multiple LLMs in triage using a large retrospective dataset. **Methods:** We conducted a retrospective analysis of 39,375 anonymized patient cases from the ED of AHEPA University General Hospital, Thessaloniki, Greece (June 2024–July 2025), extracted from the hospital’s electronic medical record system. All cases were triaged in real time according to the Emergency Severity Index (ESI) by 25 emergency physicians. In cases of uncertainty, a senior emergency physician was consulted. Seven LLMs (ChatGPT-5 Thinking, ChatGPT-5 Instant, Gemini 2.5, Qwen 3, Grok 4.0, Deep Seek v3.1, and Claude Sonnet 4) were evaluated against the physician-assigned ESI level (reference standard). Outcomes included triage score agreement (quadratic weighted kappa, κw), clinic referral accuracy and admission prediction. Subgroup analyses were performed by referral clinic and admission outcome. The study was conducted in accordance with TRIPOD-AI reporting guidelines. **Results:** Model performance varied substantially. DeepSeek and Claude Sonnet 4 achieved the highest agreement with physician-assigned ESI (κw ≈ 0.467; raw accuracy: 61.7%). In contrast, GPT-5 Instant performed poorly across all evaluation metrics (κw = 0.176; 95% CI: 0.167–0.186). Claude Sonnet 4 demonstrated the best performance in clinic referral (67.1%; κ = 0.619) and admission prediction (κw ≈ 0.46). Subgroup analyses indicated higher performance in pediatric cases and organ-specific complaints, such as ophthalmology (up to 81% accuracy). LLMs also showed tendencies toward over- or under-triage. **Conclusions:** Current LLMs demonstrate promising but inconsistent capability in triage. While selected models achieved moderate alignment with physician ESI decisions, none achieved strong agreement (κ > 0.80). LLMs are most suitable as supervised decision support tools, particularly in anatomically well-defined clinical scenarios, rather than as autonomous systems.

## 1. Introduction

Emergency departments (EDs) rely on triage scoring as a core function to prioritize patient treatment based on medical urgency [1]. Accurate triage assessments are crucial for ensuring safe patient care and the appropriate allocation of medical resources [2]. Overcrowding in EDs represents a major challenge, increasing the risk of delayed care, mis-triage and suboptimal patient outcomes [3]. To address these challenges, numerous studies have investigated strategies for improving triage training and refining decision-making processes [1,4].

The Emergency Severity Index (ESI) is a widely adopted five-level triage system designed to categorize patients based on both clinical acuity and anticipated diagnostic and therapeutic resource utilization [5]. Although different triage systems are used worldwide, no ideal triage system has yet been established [6]. Several studies have shown that trained clinicians can accurately assign triage levels, contributing to improved patient flow and better management of ED overcrowding [3,7]. To address overcrowding, various technological tools have been explored as supportive tools in triage. For instance, e-kiosks have been studied to improve pre-triage waiting times, and mobile applications were also explored as supportive tools in triage decision making [8,9]. Consequently, there is growing interest in whether other data-driven tools, such as large language models (LLMs), can support accurate and consistent triage decisions, which may improve patient safety in the ED. Moreover, disparities in triage outcomes exist across sociodemographic groups, with minority patients (such as Black and Hispanic individuals) often receiving less acute triage scores than White patients, highlighting the need for unbiased decision support tools [10,11].

LLMs offer strong computational support for clinical decision making, enabling physicians to address complex diagnostic and therapeutic challenges [12]. Seven LLMs were included in this study: ChatGPT-5 (Thinking mode), ChatGPT-5 (Instant mode), DeepSeek, Claude Sonnet 4, Qwen, Grok, and Gemini 2.5. These models differ in architecture, reasoning style, and processing capabilities, which may influence their performance in clinical decision-making tasks [13,14,15,16,17]. Their flexible architectures allow them to process unstructured clinical data, medical guidelines and diverse biomedical sources with increasing precision when multiple data types and complex patient information must be considered [18,19]. Initial assessments of LLMs show both potential benefits and current limitations. In precision oncology, LLMs frequently generated useful complementary ideas and showed notable potential in tasks such as rapidly filtering the biomedical literature to support evidence-based therapeutic planning [20]. Similarly, evaluations of GPT-3.5 and GPT-4 across real-world clinical scenarios revealed that while LLMs could provide reasonable suggestions for diagnostic steps, examinations, and treatments, they struggled most with proposing accurate initial diagnoses [21].

Current research on LLM applications in triage demonstrates considerable promise; however, reliability and accuracy still lag behind experienced clinicians [17]. Several studies report tendencies for LLMs to overestimate severity and urgency, increasing false-positive emergency classifications [22,23], while others indicate potential underestimation of clinical risk [22]. Limitations of existing studies include small sample sizes (124–4000 patients), assessment of single triage scales, and evaluation of only a few LLMs, making it difficult to derive reliable and broadly generalizable conclusions [22,23,24,25,26,27,28].

This study aims to provide a comprehensive, multidimensional evaluation of the diagnostic performance of existing LLMs in emergency triage using the ESI. The large clinical sample supports a more robust and generalizable assessment of model capabilities, helping to overcome key limitations observed in prior research.

## 2. Materials and Methods

### 2.1. Study Design and Population

We retrospectively compiled 39,375 emergency cases from 1 June 2024 to 21 July 2025 in the interdisciplinary ED of AHEPA University General Hospital (American Hellenic Educational Progressive Association), Thessaloniki, Greece. The study was conducted in accordance with TRIPOD-AI reporting guidelines [29]. The sample size of 39,375 cases exceeds those used in prior research on LLMs in triage, providing sufficient statistical power to robustly evaluate model performance in triage decisions [22,23,24,25,26,27,28].

All cases were extracted from the hospital’s electronic medical records and converted into standardized, anonymized English-language case vignettes for model evaluation. All presented symptoms and vital signs were recorded as free text in the hospital’s electronic medical record. These fields did not contain personally identifying information. Moreover, direct identifiers such as patient names, medical record numbers and dates of birth were removed prior to vignette creation. This manual review ensured that all vignettes were fully anonymized and safe for further analysis.

Patient ages ranged from 0 to 106 years, and patients of both sexes were represented. Only age and presenting symptoms were included in the LLM evaluations; vital signs and level of consciousness were included when documented at triage. Predictors (age, presenting symptoms, vital signs and level of consciousness when documented) were used in their raw form. No rescaling or standardization was applied. Missing data were handled using the available information only, without imputation. This was a retrospective observational study of triage decisions at ED presentation; no follow-up was conducted.

All patient records were originally triaged in real time according to ESI by 25 physicians, who receive ESI training every year. In cases of uncertainty, a senior emergency physician was consulted.

Inclusion and exclusion criteria were defined as follows. All ED encounters during the study period were eligible. For each model, cases were excluded from analysis if the model output was invalid (e.g., non-existent clinic names, responses outside the expected format, or outputs not mapping to predefined hospital categories). Exclusion rates varied across models, ranging from 2.1% (Thinking GPT-5, n = 839) to 9.6% (DeepSeek, n = 3794), yielding analyzable samples of 35,581–38,536 cases per model. These exclusions were reported descriptively as an indicator of output-format compliance (instruction-following robustness). No official recording of ethnicity or other sociodemographic characteristics beyond age and sex was available in the hospital records; therefore, potential disparities across ethnic or demographic groups could not be directly assessed.

### 2.2. Large Language Models Evaluated

Seven LLMs were evaluated: ChatGPT-5 (Thinking mode), ChatGPT-5 (Instant mode), DeepSeek, Claude Sonnet 4, Qwen, Grok, and Gemini 2.5. The rationale for model selection was to assess a range of widely available LLMs in a real-world triage scenario. Models were accessed during the study period using their publicly available interfaces with default generation parameters. No additional training or fine-tuning was performed. All LLMs were evaluated in their default, publicly available configurations, without any update or recalibration. Each model was evaluated on the full set of eligible cases.

### 2.3. Prompt Design for LLMs

A single, fixed prompt was used for all models to ensure consistency and comparability across evaluations. The prompt was not modified or optimized for individual models, reflecting a real-world, survey-style deployment rather than model-specific fine-tuning.

The following instruction was provided to all LLMs:

“I will provide you with clinical information regarding specific patients, including their age and the symptoms with which they presented to the emergency department. Based on these data, I would like you to generate a structured summary containing specific elements in a predetermined order. More precisely, I require you to carefully apply the distinctions and decision-making algorithms of the five-level ESI triage system, and to calculate the corresponding score for each patient. Subsequently, you should determine the most appropriate referral clinic, which must be written in Greek capital letters. Finally, you should indicate whether hospital admission is required, using 1 to denote the need for admission/hospitalization and 0 to denote that admission is not required. The results must appear strictly in the following order: ESI—Referral Clinic—Admission Prediction, and they should be presented in Excel-compatible format to facilitate further data processing. The available referral clinics are: Vascular Surgery, Cardiology, Cardiac Surgery, Neurology, Neurosurgery, Nephrology, Orthopedics, Pathology, Surgery, Psychiatry, Otolaryngology, Ophthalmology, Pediatrics, Fast-Track, Shock-Room. You should select the most appropriate one for each case and provide it in Greek capital letters.”

### 2.4. Triage Systems

Each case was evaluated by all seven LLMs using the five-level ESI triage system [5]. The ESI results of the LLMs were directly compared to physician-assigned ESI levels to assess agreement. At the time of triage, physicians were able to measure all vital signs and assess level of consciousness; however, these parameters were not documented for every patient. Consequently, LLMs were provided exclusively with information recorded at triage. When vital signs or level of consciousness were measured by the triage physician, they were documented and made equally available to the LLMs, ensuring comparable clinical information for both human and AI assessments. Vital signs were documented for the majority of encounters; missing vital signs occurred mainly in a small subset of clearly low-acuity presentations in which full vital sign measurement was not routinely performed. Recorded vital signs included heart rate, respiratory rate, oxygen saturation, and, when available, blood pressure, temperature, and glucose.

### 2.5. Ethical Considerations

This study was conducted in accordance with the Declaration of Helsinki and was approved by the local ethics committee (approval no. 212/23 May 2025). Due to its retrospective design, all data were anonymized, and informed consent was not required. Patients and the public were not involved in the design, conduct, reporting, interpretation or dissemination of this study.

### 2.6. Statistical Analysis

Statistical analysis evaluated agreement between physician triage decisions and LLM predictions for the ESI five-level triage scale. Quadratic weighted Cohen’s kappa (κ_w_) was used, as it penalizes large disagreements (e.g., confusing Level 1 with Level 5) more heavily than minor discrepancies [30,31]. For binary admission decisions, standard Cohen’s kappa was calculated. Classification performance was evaluated using accuracy, sensitivity and specificity, as well as F1-scores where appropriate. For multiclass clinic referral, the weighted F1-score was used to account for class imbalance across specialties. Agreement for the 15-specialty clinic referral was assessed using multiclass Cohen’s kappa analysis. Confusion matrices were constructed to illustrate patterns of errors between physicians and LLMs. McNemar’s test was used to compare LLM predictions against physician-assigned admission decisions. Agreement strength was interpreted as: <0.20—poor, 0.21–0.40—fair, 0.41–0.60—moderate, 0.61–0.80—substantial and 0.81–1.00—almost perfect [32]. Additional sensitivity analyses comparing top-performing models using paired bootstrap resampling were conducted.

To assess whether small observed differences in agreement reflected systematic performance variation rather than sampling noise, we conducted a paired bootstrap analysis among the three top-performing models identified in the primary analyses (Claude Sonnet 4, DeepSeek, and Gemini 2.5). Analyses were restricted to cases with valid outputs from all three models to ensure identical patient samples. Bootstrap resampling preserved within-case pairing across models.

### 2.7. Sub-Analysis of Clinical Features

Prespecified subgroup analyses were conducted to explore variation in model performance across clinically relevant strata. Performance metrics were stratified by referral clinic and admission outcome.

## 3. Results

### 3.1. Triage Score Agreement

**Figure 1:**
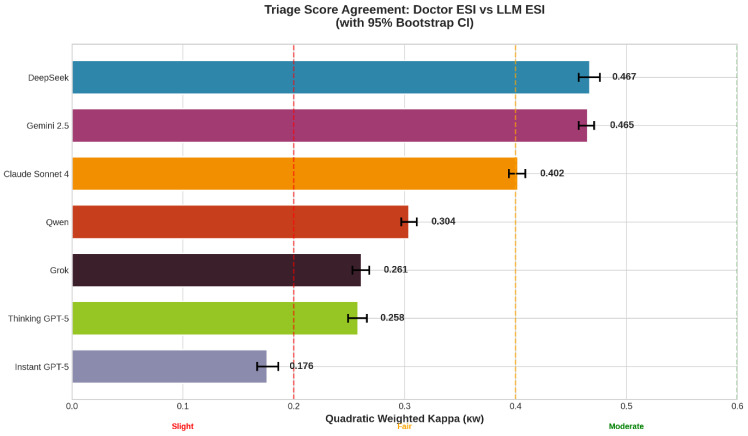
Agreement between LLM and physician triage scores. Quadratic weighted kappa (κ_w_) for each model compared to the physician ESI score. Error bars represent 95% confidence intervals.

From 39,375 emergency department encounters, valid LLM responses were analyzed for concordance with physician-assigned ESI triage levels. Figure 1 summarizes agreement using quadratic weighted Cohen’s kappa (κ_w_) for each model. DeepSeek demonstrated the highest agreement with physician ESI assessments (κ_w_ = 0.467; 95% CI: 0.457–0.476), followed closely by Gemini 2.5 (κ_w_ = 0.465; 95% CI: 0.457–0.471). Both models achieved moderate agreement. Claude Sonnet 4 showed slightly lower agreement (κ_w_ = 0.402; 95% CI: 0.394–0.409), remaining at the boundary between fair and moderate concordance.

Qwen (κ_w_ = 0.304; 95% CI: 0.297–0.311), Grok (κ_w_ = 0.261; 95% CI: 0.253–0.268), and Thinking GPT-5 (κ_w_ = 0.258; 95% CI: 0.249–0.266) demonstrated fair agreement, while Instant GPT-5 showed poor agreement with physician triage decisions (κ_w_ = 0.176; 95% CI: 0.167–0.186).

Across all models, negative mean bias values indicated a general tendency toward over-triage relative to physician assessments; exact agreement percentages are provided for descriptive comparison (Table 1).

### 3.2. Clinic Referral Accuracy

**Figure 2:**
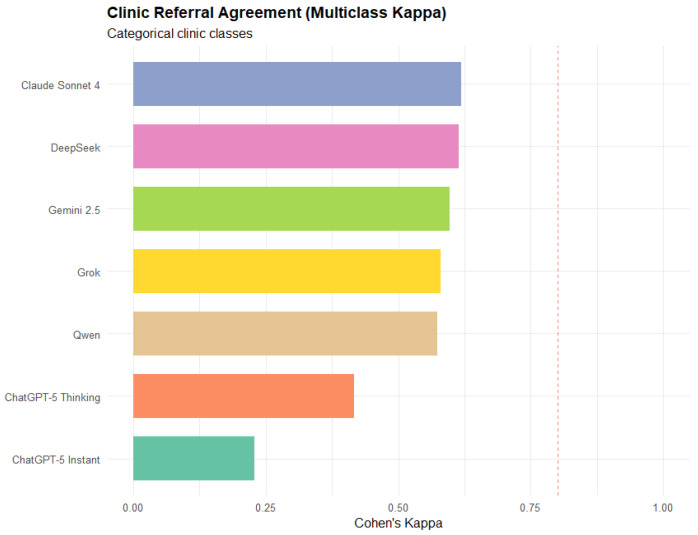
Multiclass Cohen’s kappa coefficients for clinic referral agreement. The red dashed line shows the threshold for strong agreement, which is κ > 0.80.

LLM performance in clinic referrals was evaluated across 15 specialty categories using accuracy and multiclass Cohen’s kappa. Claude Sonnet 4 achieved the highest agreement with physician referral decisions (accuracy: 67.1%; κ = 0.619, 95% CI: 0.614–0.624), corresponding to substantial agreement (Figure 2). DeepSeek demonstrated comparable performance (accuracy: 66.8%; κ = 0.615, 95% CI: 0.608–0.620), followed by Gemini 2.5 (accuracy: 64.5%; κ = 0.597, 95% CI: 0.591–0.602) and Grok (accuracy: 63.8%; κ = 0.580, 95% CI: 0.575–0.586).

Thinking GPT-5 showed moderate agreement (κ = 0.416, 95% CI: 0.411–0.423), while Instant GPT-5 demonstrated poor performance (κ = 0.229, 95% CI: 0.224–0.235). No model reached the threshold for strong agreement (κ > 0.80).

**Figure 3:**
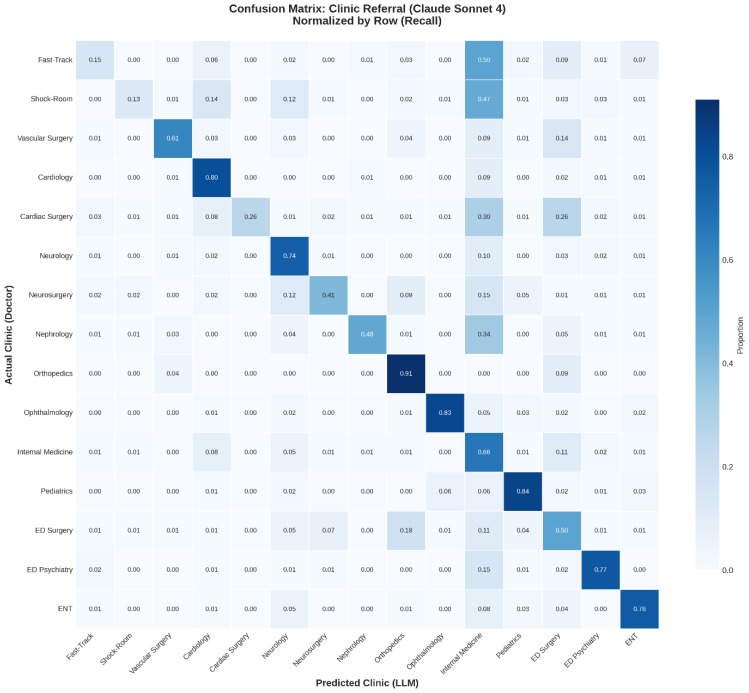
Confusion Matrix for Clinic Referral (Claude Sonnet 4). Heatmap showing the distribution of predicted vs. physician-assigned clinic destinations for Claude Sonnet 4.

A representative confusion matrix for the highest-performing model (Claude Sonnet 4) is shown in Figure 3. Correct classifications clustered along the diagonal, with strong performance in anatomically well-defined specialties such as Ophthalmology and Pediatrics. In contrast, lower recall for severity-based categories, such as Fast Track and Shock Room, was shown, with a substantial proportion of cases misclassified as Internal Medicine.

### 3.3. Performance by Clinical Specialty

Performance varied substantially across clinical specialties. Severity-based routing categories, including Fast Track and Shock Room, demonstrated the highest misclassification rates, with F1-scores below 0.25. In contrast, anatomically well-defined specialties showed consistently higher performance.

**Figure 4:**
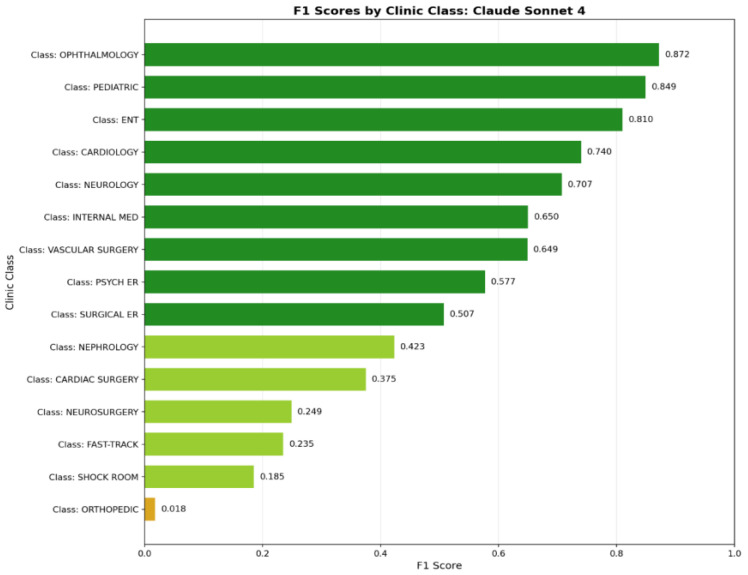
F1-scores by clinical specialty (Claude Sonnet 4).

For the highest-performing model (Claude Sonnet 4), classification performance was strongest in Ophthalmology (F1 = 0.872), Pediatrics (F1 = 0.849), and Otolaryngology (ENT; F1 = 0.810). Moderate performance was observed in Cardiology (F1 = 0.740), Neurology (F1 = 0.707), and Internal Medicine (F1 = 0.650) (Figure 4).

Performance was poorest in Orthopedics (F1 = 0.018), Shock Room (F1 = 0.185), and Fast Track (F1 = 0.235), highlighting persistent challenges in severity-based referral categories.

### 3.4. Admission Prediction (Outcome)

**Figure 5:**
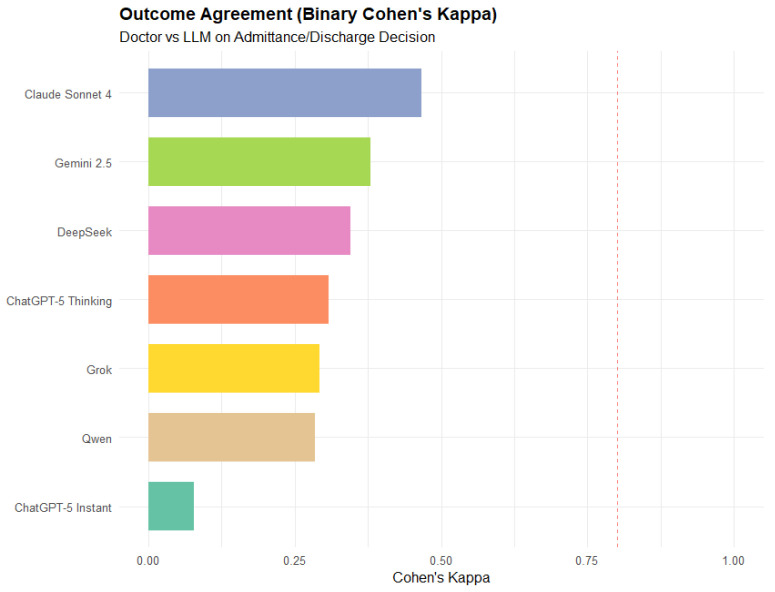
Agreement between LLM-predicted and physician-assigned admission decisions, measured using binary Cohen’s kappa. The dashed line indicates κ = 0.80.

Claude Sonnet 4 was the best-performing model, achieving a binary Cohen’s kappa of about 0.46 (Figure 5), which signifies that the model’s predictions were moderately reliable. The next best were Gemini 2.5 and DeepSeek, with almost the same performance (κ ≈ 0.37). ChatGPT-5 Instant showed the poorest result (κ < 0.10), implying that its predictions were almost random with respect to the physicians’ decisions about admissions.

### 3.5. Error Bias Analysis (McNemar’s Test)

**Figure 6:**
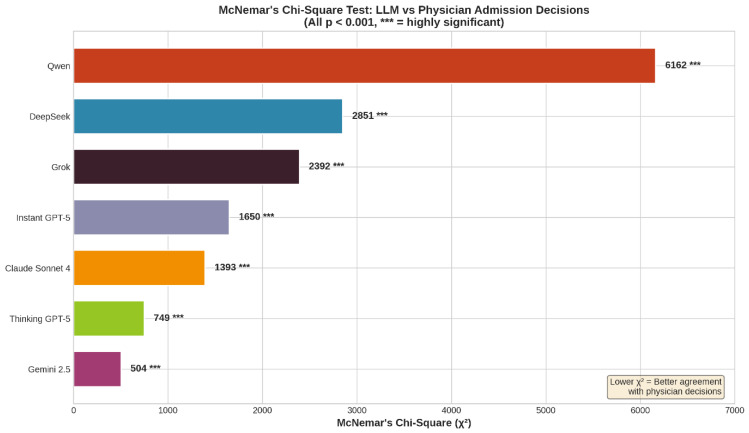
Analysis of systematic error bias (McNemar’s test). The chi-squared (χ^2^) values quantify the asymmetry of errors (false positives vs. false negatives).

McNemar’s test revealed statistically significant discordance in error patterns for all models (*p* < 0.001). However, the direction of bias varied critically. Qwen and DeepSeek exhibited a strong positive bias (χ^2^ = 6162 and 2851), generating 3–5 times more false admissions than missed admissions. In contrast, Thinking GPT-5 showed a dangerous negative bias (χ^2^ = 749), significantly favoring false discharges (missed admissions) over false positives. Gemini 2.5 demonstrated the most balanced error profile, though it still leaned toward over-admission (Figure 6).

Analysis of sensitivity and specificity revealed distinct admission decision profiles across models. Claude Sonnet 4 demonstrated the most balanced performance, maintaining comparable sensitivity (≈75%) and specificity (≈76%).

**Figure 7:**
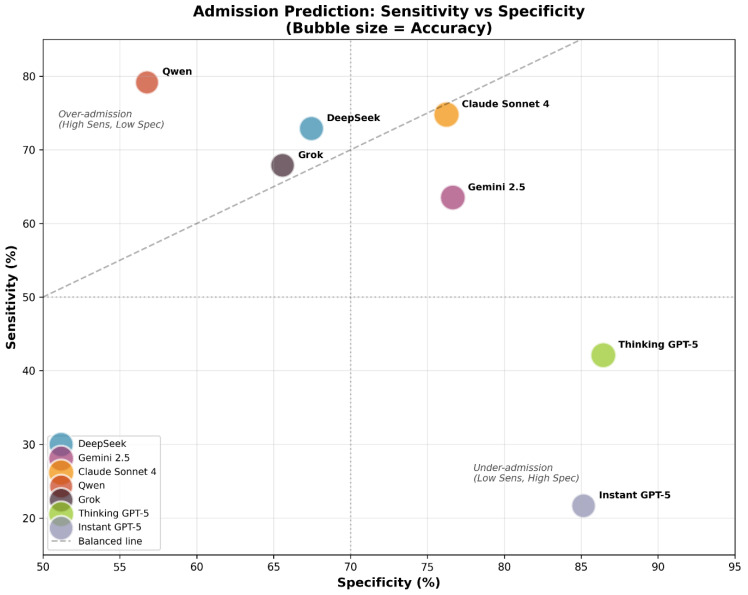
Hospital admission prediction metrics. Comparative bar chart of sensitivity (recall of admitted patients) vs. specificity (correct discharge).

In contrast, Instant GPT-5 showed markedly low sensitivity (<25%) despite high specificity (≈85%), indicating a strong tendency toward discharge decisions. Qwen exhibited the opposite pattern, achieving high sensitivity (≈79%) but substantially lower specificity (≈57%), consistent with a tendency toward over-admission (Figure 7).

These trade-offs contextualize the overall agreement results and the systematic error asymmetries identified by McNemar’s test.

McNemar’s test demonstrated statistically significant asymmetry in admission decision errors for all evaluated models (*p* < 0.05), indicating systematic differences between false-positive and false-negative predictions. The magnitude and direction of this asymmetry varied across models. Qwen exhibited the largest imbalance (χ^2^ > 6000), reflecting a strong tendency toward over-admission. In contrast, Gemini 2.5 and Thinking GPT-5 showed the smallest chi-square values, indicating more balanced false-positive and false-negative error distributions relative to physician decisions.

### 3.6. Paired Bootstrap Comparison of Top-Performing Models

To assess whether the small observed differences in agreement among the top-performing models reflected systematic performance variation rather than sampling noise, we conducted paired bootstrap analyses on matched patient samples with valid outputs from all three models (Claude Sonnet 4, DeepSeek, and Gemini 2.5) (Table 2). For ESI triage, DeepSeek demonstrated significantly higher agreement with physician-assigned scores compared with both Claude Sonnet 4 and Gemini 2.5. In contrast, admission prediction performance was significantly higher for Claude Sonnet 4 compared with both DeepSeek and Gemini 2.5. Differences in clinic referral agreement between models were small and not consistently statistically significant. These findings indicate that, while overall performance among the leading models was broadly comparable, specific strengths varied by task.

## 4. Discussion

In this large retrospective study, we evaluated the clinical decision-making performance of seven LLMs across hospital admission prediction, clinic referral selection, severity categorization, and triage scoring according to the ESI. Using a real-world dataset of 39,375 cases—one of the largest sample sizes assessed to date [23,24,25,27,28]—we observed substantial variability across models.

Overall, Claude Sonnet 4 and DeepSeek achieved the highest and most consistent agreement with physician decisions, though overall agreement remained moderate. These findings align with prior reports showing high accuracy in clinical scenarios using Claude 3.5 Sonnet [25]. In contrast, Instant GPT-5 consistently underperformed, showing low accuracy, weak kappa values, and limited stability across all tasks. These differences likely reflect variations in model architecture and reasoning strategies, consistent with prior studies demonstrating that reasoning optimized modes enhance diagnostic accuracy [33].

Triage and specialty-specific performance varied notably. LLMs performed best in domains with clearly anatomical or organ-specific patterns—ophthalmology, pediatrics and ENT cases—approaching near-specialist-level accuracy. The higher performance in these domains likely reflects distinct symptomatology and vocabulary that facilitate pattern recognition, a phenomenon also observed in the study by Lyons et al. [34]. However, scenarios requiring severity-based assessment—shock room, fast track and orthopedics—posed significant challenges. These scenarios require synthesis of high-impact clinical signs, such as abnormal vital signs and elements of clinical gestalt, as well as contextual reasoning that remains challenging for current LLMs [35]. In our study, many of these critical cues were not consistently documented across all cases. As ESI depends heavily on such inputs, this likely constrained model performance in severity-based categories. Consequently, when evaluated primarily on inputs lacking structured clinical data, LLMs may struggle to accurately classify such cases. Additionally, the orthopedic category should be interpreted with caution, as our hospital does not have a dedicated orthopedics department and only manages a small number of urgent cases. Misclassification in these cases carries significant critical risk; for example, delayed recognition of severe conditions like acute limb ischemia can increase morbidity and mortality [36]. These findings suggest that current LLMs should be used with caution in severity-driven decision making. Providing more detailed clinical information for shock room and fast track cases could potentially improve LLM’s severity-related predictions.

Admission prediction revealed similar trends across models. Claude Sonnet 4 achieved moderate predictive power (κ ≈ 0.46, sensitivity: ~75%, specificity: ~76%). Other models displayed substantial biases. Instant GPT-5 exhibited extremely low sensitivity (<25%), with high specificity (~85%), indicating under-triage risk. In emergency medicine, missed admissions and underestimation of clinical severity have been associated with increased mortality, making this pattern particularly concerning [37]. In contrast, Qwen and Grok tended to over-triage patients (high sensitivity: ~79%, low specificity: ~57%). This finding is consistent with previous studies, which have reported that LLMs tend toward over-triage [22,23]. These opposing biases indicate the need for careful calibration and validation of individual models before any clinical integration and also raise ethical concerns [37]. The recent literature emphasizes that inaccurate or biased LLM outputs may affect patient safety, influence care decisions and lead clinicians to over-rely on model suggestions [38,39].

The comparison between GPT-5 variants further illustrates the importance of structured reasoning. Thinking mode consistently outperformed across all metrics (triage scoring, clinic referral accuracy, and admission prediction). This aligns with prior research showing that chain-of-thought and structured reasoning prompts improve performance in complex clinical tasks [33,40]. However, no model achieved strong agreement with clinicians (κ > 0.80), emphasizing that current LLMs lack the reliability required for independent triage decision making. This limitation is consistent with the broader artificial intelligence (AI) literature, which highlights challenges related to confabulation, limited contextual awareness and biases inherited from training data [41].

From a clinical perspective, current LLMs may offer value as supportive tools rather than autonomous decision-making tools in triage. Our analysis shows that performance differences among top-performing models were systematic and task-dependent, meaning that no single model consistently outperformed others across all clinical decisions. This highlights that their main benefit lies in augmenting clinical expertise rather than replacing it. LLMs may also help mitigate ED overcrowding by optimizing time and resource allocation [42]. Evidence suggests that physicians are willing to modify clinical decisions based on LLM assistance in standardized chest pain scenarios [43]; however, erroneous recommendations can propagate automation bias and degrade performance [44,45]. Strategies such as retrieval-augmented generation, combining multiple LLMs to reduce model-specific biases, and clinician review may reduce these risks [25,40,46].

This study has limitations. This was a single-center retrospective study in which some text-based inputs did not consistently include vital signs or level of consciousness. Although the ESI formally requires vital signs for triage, incomplete documentation occurred in a minority of cases and reflects real-world clinical practice. This inconsistent documentation of critical cues may contribute to lower ESI assessment reliability in our study. Our hospital does not have a dedicated orthopedics department and only manages a small number of urgent cases, so the orthopedic category should be interpreted with caution. Sample sizes varied across LLMs (range: 35,581–38,536; exclusion rates: 2.1–9.6%) due to invalid model outputs. However, exclusions were based on predefined validity criteria (non-conforming clinic names or output formats) rather than case characteristics, minimizing the risk of selection bias. Additionally, LLM performance is sensitive to prompt design and model version update, which limits reproducibility over time. Finally, the tested LLMs were generalist models and had not been fine-tuned for health applications. This likely contributed to lower ESI assessment reliability in our study.

Future research should evaluate fine-tuned and multimodal LLM architectures and assess their performance across multiple healthcare settings. Moreover, prospective studies incorporating structured clinical inputs should assess actual patient outcomes when LLMs are part of triage workflows. These studies may further support a “triage assistant” role in real-world settings.

## 5. Conclusions

In summary, across seven models, performance varied substantially. While selected models demonstrated moderate alignment with physician ESI decisions and consistent performance in clinical referral and admission decisions, none achieved high-level concordance suitable for autonomous triage. LLMs performed more reliably in anatomically defined scenarios and pediatric cases but struggled with severity-based triage. These findings support the use of LLMs as adjunctive tools under clinician supervision rather than autonomous systems in triage.

## Figures and Tables

**Table 1:** Triage score agreement and bias analysis. Comparison of quadratic weighted Cohen’s kappa and mean bias across seven LLMs. Negative bias values correspond to higher triage scores assigned by the model; 95% confidence intervals (CIs) were calculated via bootstrap resampling.

Model	N	κ_w_	95% CI	Exact Agreement (%)	Bias	Interpretation
DeepSeek	35,581	0.467	0.457–0.476	59.4%	−0.22	Moderate
Gemini 2.5	38,410	0.465	0.457–0.471	43.6%	−0.38	Moderate
Claude Sonnet 4	37,897	0.402	0.394–0.409	48.0%	−0.46	Fair
Qwen	36,372	0.304	0.297–0.311	36.7%	−0.67	Fair
Grok	36,585	0.261	0.253–0.268	34.2%	−0.74	Fair
Thinking GPT-5	38,536	0.258	0.249–0.266	39.5%	−0.26	Fair
Instant GPT-5	37,884	0.176	0.167–0.186	40.1%	−0.15	Slight

κ_w_ = quadratic weighted kappa; CI = confidence interval; bias = mean deviation. Agreement interpretation: <0.20—slight, 0.21–0.40—fair, 0.41–0.60—moderate, 0.61–0.80—substantial, >0.80—almost perfect.

**Table 2:** Paired bootstrap comparison of top three LLMs.

Comparison	Δκ	95% CI	*p*-Value
**Triage Score (Quadratic Weighted Kappa, κw)**			
Claude Sonnet 4 vs. DeepSeek	−0.074	[−0.085, −0.063]	<0.001
Claude Sonnet 4 vs. Gemini 2.5	−0.059	[−0.068, −0.050]	<0.001
DeepSeek vs. Gemini 2.5	+0.015	[+0.005, +0.025]	0.005
**Clinic Referral (Multiclass Kappa, κ)**			
Claude Sonnet 4 vs. DeepSeek	−0.003	[−0.010, +0.005]	0.494
Claude Sonnet 4 vs. Gemini 2.5	+0.006	[−0.001, +0.013]	0.104
DeepSeek vs. Gemini 2.5	+0.008	[+0.001, +0.016]	0.020
**Admission Prediction (Binary Kappa, κ)**			
Claude Sonnet 4 vs. DeepSeek	+0.113	[+0.101, +0.125]	<0.001
Claude Sonnet 4 vs. Gemini 2.5	+0.091	[+0.078, +0.104]	<0.001
DeepSeek vs. Gemini 2.5	−0.022	[−0.035, −0.009]	<0.001

κ_w_ = quadratic weighted kappa; CI = confidence interval; Δκ = delta kappa.

## Data Availability

The data underlying this article will be shared upon reasonable request by the corresponding author.

## Abbreviations

The following abbreviations are used in this manuscript:

ED	Emergency Department
ENT	Ear, Nose, and Throat (Otolaryngology)
LLM	Large Language Model
ESI	Emergency Severity Index
F1	F1-score (harmonic mean of precision and recall)
κ	Cohen’s Kappa (coefficient for inter-rater agreement)
κ_w_	Quadratic Weighted Cohen’s Kappa
χ^2^	Chi-squared statistic
